# Miscellany

**Published:** 1891-09

**Authors:** 


### Miscellany.

---

MEDICAL SOCIETY OF THE STATE OF NEW YORK.—The Secretary is authorized to offer to the County Societies, and to other Societies which send delegates to this Society, such of the back volumes of the *Transactions* as are in his hands, to complete, so far as they may, their files of this publication. For the information of such Societies as may wish to avail themselves of this, he would state that at present (1891) there are on hand *Transactions* of the following years:

| | |
|---|---|
| 1807–31 (Reprint in one volume.) | 1861 |
| 1840—43 (Reprint in one volume.) | 1864 |
| 1860 | 1868 |

and all succeeding years to the present time; of some, the number of copies on hand is limited.

Any of these issues that may be lacking will be sent gratuitously to all Societies that have already subscribed for their quotas (five copies to each delegate sent) for past years, or that will subscribe for their quotas for 1890 and 1891 if they have not already done so. It is thought very desirable that each Society should provide itself with as complete a file as possible of these volumes, and preserve them carefully in its own possession. Some of them cannot be easily replaced, and a complete set is practically a history of medicine in this State for this century. Some County Societies that have subscribed for them have failed to preserve complete sets, and some have not subscribed regularly; it is to be hoped that they may all do so hereafter. Societies receive their quotas at $1.25 per volume; to others the price is $1.50, and, for issues prior to 1887, fifty cents.

Requests for volumes wanted as above set forth may be sent to the Secretary, Dr. F. C. Curtis, 17 Washington avenue, Albany, or the Treasurer, Dr. C. H. Porter, 103 Lancaster street, Albany.

The American people are undoubtedly to-day the greatest reading people in the world. Even in very humble households you will find a magazine, one or two weekly papers, and a half dozen shelves of books; the cabinet maker furnishing in these days a very elegant oak book-case for $8.00. There are three classes of publications which are found in almost every household—books, the weekly paper, and the monthly illustrated magazine. The weekly paper gives all the news of the week, keeps the subscriber posted upon what is going on in the markets and in politics. The illustrated magazine, owing to the fact that it is seldom published in numbers less than 100,000, can afford to pay the highest prices for both literary and artistic work, and offers at a figure so low that it seems impossible, the serious, sober thoughts of the great writers of the world, in addition to those of masters of fiction, together with lighter and more entertaining sketches.

When the public was first presented with an illustrated magazine for $4.00, and a weekly at $2.00, it was thought that great progress had been made in giving much for little. What must be thought then of a proposition which gives a magazine like the *Cosmopolitan*, the rival of the best of the $4.00 magazines, a monthly of sixty-four pages, closely printed like the BUFFALO MEDICAL AND SURGICAL JOURNAL for $2.00, and then, to cap the climax, throws in for 50 cts. the publishers' regular subscription edition of a copyright work like Grant's Memoirs, sold at $7.00; not a cheap reprint, but on the best paper, handsomely bound in cloth, green and gold, in every respect the same edition as has hitherto been sold at $7.00—$5.00 (not including postage on Memoirs) for the entire combination. Such an offer constitutes an event in the history of book publishing. It has never been done before, and probably will never be repeated, unless some of the older magazines should likewise aspire to build up their circulation to half a million copies.

